# Plasmon-Enhanced Copper Nanodendrites as Dual-Function SERS Substrates and Photoelectrochemical H_2_O_2_ Sensors

**DOI:** 10.3390/nano16191269

**Published:** 2026-10-08

**Authors:** Ying-Chu Chen, Cing-Jhen Shih, Yu-Kuei Hsu

**Affiliations:** 1 Department of Chemical Engineering and Biotechnology, National Taipei University of Technology, No. 1, Sec. 3, Zhongxiao E. Road, Taipei 10608, Taiwan; 2 Department of Opto-Electronic Engineering, National Dong Hwa University, No. 1, Sec. 2, Da Hsueh Road, Shoufeng, Hualien 97401, Taiwan

**Keywords:** copper nanodendrites, localized surface plasmon resonance, surface-enhanced Raman scattering, photoelectrochemical sensing, hydrogen peroxide

## Abstract

A facile, one-step galvanic replacement route was used to grow hierarchical copper nanodendrites (Cu NDs) directly on aluminum (Al) foil, providing a dual-function plasmonic platform for surface-enhanced Raman scattering (SERS) and enzyme-free photoelectrochemical (PEC) detection of hydrogen peroxide (H_2_O_2_). The Cu NDs were formed by immersing Al foil in an acidified CuSO_4_ solution at room temperature, and their fern-leaf morphology, phase purity, and metallic Cu(0) oxidation state were confirmed by scanning electron microscopy, X-ray diffraction, and X-ray photoelectron spectroscopy. Using 4-mercaptobenzoic acid (4-MBA) as a probe molecule, the Cu NDs/Al foil substrate achieved SERS detection down to 10^−7^ M with an approximate enhancement factor of ~10^6^, consistent with strong localized surface plasmon resonance (LSPR)-driven electromagnetic enhancement. Under simulated solar illumination, LSPR-excited hot carriers additionally accelerated the electrocatalytic reduction of H_2_O_2_, raising the detection sensitivity from 110.6 to 145.3 μA cm^−2^ mM^−1^ relative to dark conditions. Wavelength- and intensity-dependent photocurrent measurements confirmed that this enhancement tracks the LSPR absorption band of the Cu NDs rather than photothermal heating. These results establish low-cost, earth-abundant Cu nanodendrites as an effective plasmonic platform for combined optical and electrochemical (bio)sensing.

## 1. Introduction

The unique plasmonic properties of metallic nanostructures have placed them at the forefront of nanoscience. Plasmonic materials developed to date are typically based on gold and silver, whose relatively high cost and limited natural abundance hinder the large-scale commercialization of plasmonic devices, particularly for disposable or point-of-care sensing platforms. Copper (Cu) is a considerably cheaper and more earth-abundant alternative, and Cu-based nanostructures have already been explored for photonics, catalysis, nanothermometry, biosensing, and nanomedicine applications; separately, Cu-containing nanoparticles are also widely used as antibacterial and antimicrobial agents in coatings, dental materials, and other biomedical contexts, reflecting the broader practical appeal of copper as a functional nanomaterial beyond optics [1,2]. A recurring practical concern with Cu-based nanostructures, including nanowires and nanoparticles used in transparent electrodes, is their susceptibility to atmospheric oxidation, which can degrade both electrical and plasmonic performance over time unless the surface is protected or otherwise stabilized [3]. Colloidal and substrate-bound Cu nanoparticles synthesized by simple wet-chemical routes have been shown to exhibit a well-defined surface plasmon resonance (SPR) band near 570 nm, and both experimental Raman measurements and density functional theory calculations of Cu–molecule interactions confirm that this plasmonic response can meaningfully enhance the Raman signal of adsorbed probe molecules, supporting the use of Cu as a genuine alternative to noble-metal SERS substrates [4]. Semiconducting copper oxide phases (Cu_2_O, CuO) formed by simple oxidation of Cu foils or wires show a related, if generally weaker, SERS activity that combines electromagnetic enhancement with a charge-transfer contribution from the oxide, further illustrating the breadth of low-cost, Cu-based materials available for plasmon- and semiconductor-assisted Raman sensing [5]. Among the various Cu morphologies reported—plates, cubes, wires, and flowers—dendritic (fern-leaf-like) structures have attracted particular interest because their hierarchical, multi-branched architecture concentrates electromagnetic hot spots at branch tips and junctions, which is especially valuable for trace-level SERS detection. Related dendritic metal architectures grown directly on low-cost metal substrates, such as silver nanodendrites electrolessly deposited on copper plates or three-dimensional dendritic Ag nanostructures built by templated electrodeposition, have demonstrated that fern-like branching morphologies can push SERS detection limits down to trace and even ultra-trace concentrations of small organic and biological analytes [6,7]. This body of work indicates that the hierarchical dendritic morphology, rather than the specific choice of noble or non-noble metal alone, may contribute to the formation of SERS-active regions.

Cu nanodendrites (NDs) can exhibit morphology-dependent localized surface plasmon resonance (LSPR) bands in the visible–near-infrared region, which is better matched to biological sensing windows than the absorption peaks of gold (≈530 nm) and silver (≈400 nm). This strong optical response arises from the collective oscillation of free electrons in the Cu NDs—the localized surface plasmon resonance (LSPR)—which generates energetic (“hot”) carriers and locally enhances the electromagnetic field at the nanostructure surface. Recent theoretical and experimental work on plasmonic hot-electron dynamics has clarified how these non-equilibrium carriers are generated upon plasmon decay, how they are transported and injected across metal–semiconductor or metal–electrolyte interfaces, and how their short lifetimes and competition with direct photothermal heating govern the efficiency with which they can be extracted to drive a chemical or electrochemical reaction before relaxing back to equilibrium [8,9,10]. In electrochemical sensing specifically, this hot-carrier injection pathway has been exploited to accelerate a range of redox reactions: plasmonic Pd-tipped Au nanobipyramids, Au nanobipyramid–MoS_2_ core–shell hybrids, and size-tunable Ag nanoplates have all been reported to markedly enhance the electrochemical reduction of hydrogen peroxide (H_2_O_2_) under resonant illumination relative to dark conditions, with the magnitude and wavelength dependence of the enhancement tracking the LSPR absorption band of the respective nanostructure [11,12,13,14,15]. Hydrogen peroxide (H_2_O_2_) is a strong oxidant and a key reactive oxygen species that is widely used in disinfection, bleaching, environmental remediation, and chemical synthesis. It also plays important roles in biological signaling and oxidative stress, and abnormal H_2_O_2_ levels are associated with various diseases. Therefore, rapid, sensitive, and selective detection of H_2_O_2_ is important in environmental monitoring, industrial process control, and clinical diagnosis [16]. Conventional analytical methods, including titration, spectrophotometry, fluorescence, chemiluminescence, and chromatography, often require bulky instruments, complex sample preparation, or unstable enzymes. Electrochemical enzyme-free sensors offer simple operation, low cost, and rapid response, while photoelectrochemical (PEC) sensors combine light excitation with electrochemical detection to provide low background, high sensitivity, and tunable sensing performance. In particular, plasmon-enhanced PEC sensing exploits LSPR-generated hot carriers to amplify electrochemical reactions, offering a promising route to sensitive and miniaturized H_2_O_2_ detection. In this work, the LSPR response of low-cost Cu NDs is instead exploited for two complementary sensing applications: surface-enhanced Raman scattering (SERS) and photoelectrochemical (PEC) detection of H_2_O_2_.

The synthesis route used to obtain the Cu NDs is also central to their practical appeal. Our group previously reported a two-stage route to Cu NDs involving alternating electro-oxidation and chemical reduction of Cu foil; however, this process is time-consuming and adds complexity that limits practical use. Galvanic replacement offers an attractive alternative: it is a spontaneous, one-pot redox process in which atoms are oxidized and dissolved from a sacrificial metal template while ions of a more noble metal are simultaneously reduced and deposited on that same template, driven purely by the difference in standard reduction potentials between the two metals. This approach has recently been revisited as a general and versatile route to metal and bimetallic nanostructures, bridging conventional wet-chemical synthesis and electrochemical deposition methods, and has been shown to give fine control over nanostructure morphology, composition, and surface area simply by tuning the precursor concentration, reaction time, and template chemistry [17]. Related galvanic-replacement strategies have also been used specifically to decorate three-dimensional nickel and copper surfaces with metallic nanoparticles and nanostructures for electrocatalytic applications, underscoring the generality and scalability of this synthetic approach for producing hierarchical metal architectures directly on functional substrates [18]. Building on these principles, a facile one-pot synthesis of Cu NDs is achieved here by galvanic replacement, simply immersing aluminum (Al) foil in an acidified copper sulfate solution at room temperature for 1 h. The resulting dendritic Cu nanostructure on Al foil was evaluated as both a SERS substrate and an enzyme-free PEC H_2_O_2_ sensor under LSPR excitation. These low-cost, hierarchical Cu NDs/Al foil electrodes not only function effectively as SERS substrates and electrochemical electrodes but also point toward new opportunities for 3D nanostructured, plasmon-enhanced (bio)sensing platforms that do not rely on noble metals. Because both the Al foil template and the CuSO_4_ precursor are inexpensive, widely available materials, and because the entire growth step proceeds at room temperature within one hour without any external bias, capping agent, or post-synthesis reduction step, this route is inherently compatible with scalable, disposable sensor fabrication—an important practical consideration that is often secondary to sensitivity or detection limit in academic reports on noble-metal plasmonic substrates. It is also worth noting that the Al foil used here as the sacrificial template is itself a recognized, if UV-shifted, plasmonic material: Al-based nanostructures have been engineered as refractive-index sensors and ultranarrow-linewidth resonators in the ultraviolet range, a regime largely inaccessible to Cu, Ag, or Au [19,20]. In the present system the Al foil serves purely as a chemical reagent and current collector rather than as an active plasmonic component, but this dual plasmonic character of the two constituent metals is a useful point of reference for future designs that might exploit both the visible-range LSPR of the Cu NDs and the UV response of any residual or engineered Al nanostructure. In the remainder of this paper, the morphological, structural, and optical properties of the resulting Cu NDs/Al foil electrodes are first established, after which their dual performance as a SERS substrate and as an LSPR-enhanced PEC H_2_O_2_ sensor is evaluated and discussed in the context of the broader plasmonic sensing literature summarized above.

## 2. Materials and Methods

### 2.1. Synthesis of Cu NDs/Al Foil

**Figure 1:**
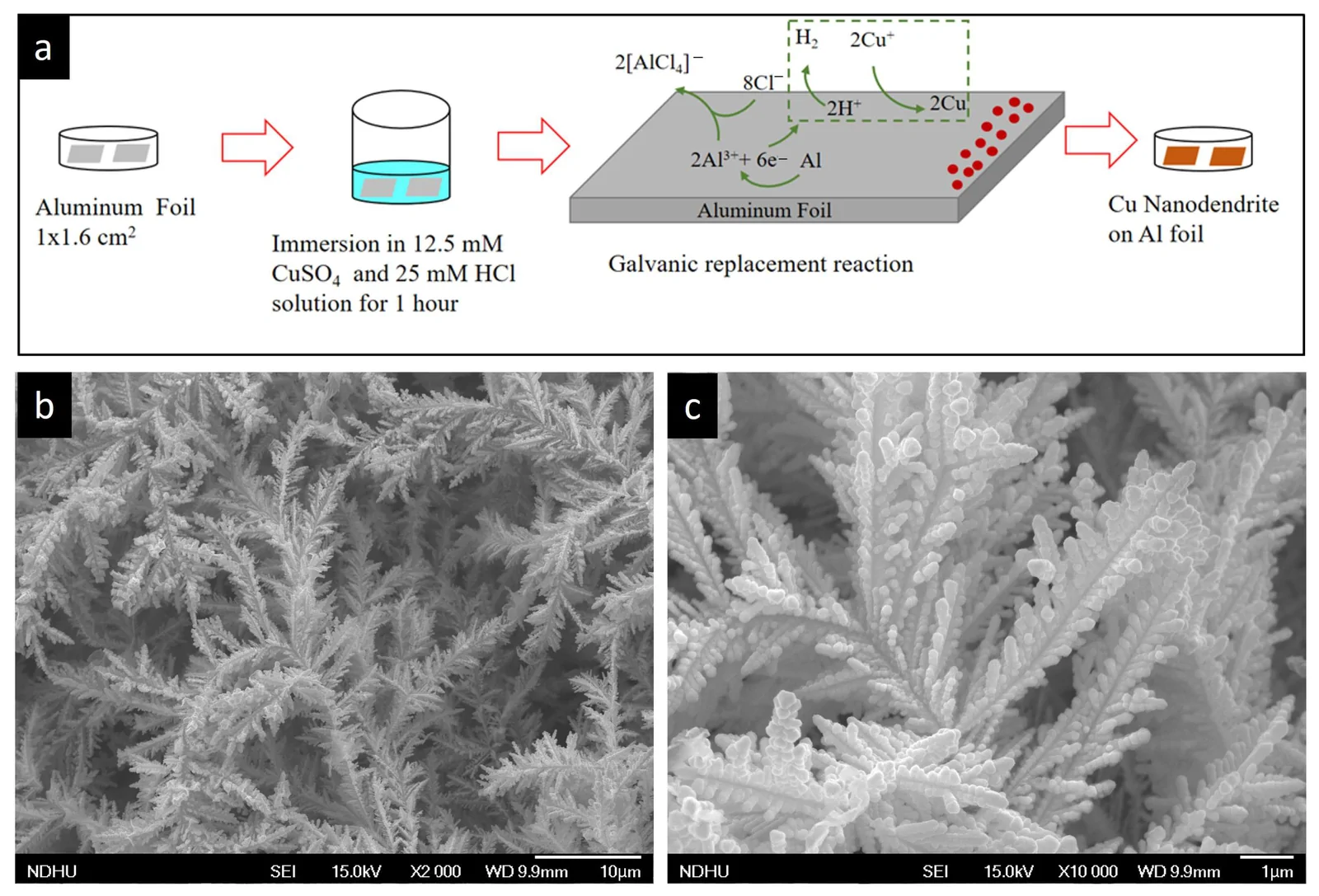
(**a**) Schematic of Cu NDs/Al foil synthesis by galvanic replacement. FESEM images of the Cu NDs at (**b**) low and (**c**) high magnification.

Cu NDs were grown on Al foil (purity 99.999%, Alfa Aesar, Ward Hill, MA, USA) via a galvanic replacement reaction, as illustrated in Figure 1a. Briefly, 12.5 mM CuSO_4_ (Sigma-Aldrich, St. Louis, MO, USA) was dissolved in 25 mM HCl aqueous solution (Sinopharm Chemical Reagent Co., Ltd., Shanghai, China). An Al foil (1 × 2 cm^2^) was cleaned with dilute hydrochloric acid, rinsed three times with deionized water (from a Milli-Q water purification system, MilliporeSigma, Burlington, MA, USA), and dried under flowing nitrogen. The Al foil was then immersed in the CuSO_4_ solution; red, metallic Cu particles formed within minutes with visible effervescence, and a dense layer of Cu NDs developed over the foil surface after 1 h. The samples were rinsed three times with deionized water and dried in a vacuum oven (DZF-6050, Shanghai Yiheng Scientific Instrument Co., Ltd., Shanghai, China) at room temperature. For SERS measurements, the samples were immersed in 4-mercaptobenzoic acid (4-MBA) (4-MBA, 99%, Tokyo Chemical Industry, Tokyo, Japan) solutions of varying concentration for 1 h, then removed and rinsed several times with deionized water. For electrochemical H_2_O_2_ detection, the Cu NDs/Al foil samples served as the working electrode in phosphate-buffered saline (PBS, 0.1 M, pH 7.4 Solarbio Life Sciences, Beijing, China) using a potentiostat/galvanostat (CHI 6273D, CH Instruments, Inc., Austin, TX, USA) in a conventional three-electrode configuration, with a platinum sheet as the counter electrode and Ag/AgCl (3 M KCl) as the reference electrode. All potentials reported herein are referenced to Ag/AgCl (3 M KCl, 0.207 V vs. SHE). All SERS and electrochemical/PEC measurements were performed immediately after synthesis. The Cu NDs/Al foil samples were rinsed quickly, kept in deionized water or the relevant measurement solution, and not exposed to air for prolonged periods before measurement. For SERS, the adsorbed 4-MBA monolayer passivates the Cu surface; for PEC measurements, the electrode was kept immersed in PBS throughout the measurement.

### 2.2. Characterization

Sample morphology was examined by scanning electron microscopy (SEM, JEM-4000EX , JEOL Ltd., Tokyo, Japan). Crystal structure was determined by X-ray diffraction (XRD, Rigaku D/Max-2500 V , Rigaku Corporation, Tokyo, Japan), and elemental chemical states were assessed by X-ray photoelectron spectroscopy (XPS , model ESCALAB Xi+, Thermo Fisher Scientific, Waltham, MA, USA). UV–vis optical spectra were measured using a Perkin-Elmer Lambda 35 spectrophotometer (PerkinElmer, Inc., Waltham, MA, USA) in reflectance mode, with bare Al foil serving as the reference substrate. To further verify the reliability and reproducibility of the optical response, additional measurements were performed using a Shimadzu UV-2600i spectrophotometer (Shimadzu Corporation, Kyoto, Japan) equipped with an integrating sphere. The spectra obtained from the two measurement systems exhibited consistent spectral profiles and resonance positions, confirming the reproducibility of the optical response of the Cu NDs/Al foil samples. Raman spectra were collected on a LabRAM HR 550 system (Horiba Scientific, Edison, NJ, USA) equipped with a thermoelectrically cooled CCD detector (spectral accuracy of 1 cm^−1^) and a 100× objective, using a He–Ne laser (632.8 nm, 5 mW , Thorlabs, Inc., Newton, NJ, USA) as the excitation source; SERS spectra were acquired with a 30 s accumulation time. Electrochemical impedance spectroscopy (EIS) was performed at 100 kHz with a 10 mV AC amplitude over a range of applied potentials. Simulated solar illumination was provided by a 150 W Xe lamp fitted with an AM 1.5G filter (Oriel Instruments, Newport Corporation, Irvine, CA, USA), calibrated to 100 mW cm^−2^. Wavelength-dependent measurements used five single-wavelength laser diodes (450, 532, 658, 808, and 980 nm , Thorlabs, Inc., Newton, NJ, USA). Losses of incident light through the quartz window and electrolyte before reaching the electrode were not corrected for.

## 3. Results and Discussion

### 3.1. Morphological, Structural, and Optical Characterization of Cu NDs/Al Foil

Figure 1b,c show the morphology of the Cu NDs grown on Al foil by galvanic replacement. The fern-leaf-like Cu NDs consist of a primary trunk 10–20 μm in length decorated with secondary branches of 0.5–4 μm; tertiary branching, where present, extends a further 0.2–2 μm (Figure 1c). The replacement reaction proceeds rapidly because of the large difference in reduction potential between the two metals (Cu^2+^/Cu, +0.34 V_RHE_; Al^3+^/Al, −1.66 V_RHE_). HCl was included in the reaction bath to suppress formation of a passivating Al_2_O_3_ layer on the Al surface; at the same time, competing H^+^ reduction to H_2_ gas moderates the rate of Cu deposition. Together, these effects allow the dense, hierarchical Cu NDs to form directly on the metallic Al substrate through a single-step chemical process. This growth behavior is broadly consistent with the general picture that has emerged from recent mechanistic studies of galvanic replacement, in which the morphology of the deposited metal is governed by the interplay between the intrinsic rate of oxidative dissolution of the sacrificial template, the rate of reductive nucleation and growth of the new metal phase, and any competing side reactions, such as hydrogen evolution, that consume charge without contributing to deposition; depending on the relative magnitude of these rates, galvanic replacement can yield anything from thin conformal shells to open, highly branched dendritic overlayers of the kind observed here [17]. The absence of any capping agent, surfactant, or applied potential in the present synthesis suggests that the fern-leaf branching morphology of the Cu NDs arises primarily from diffusion-limited growth under the locally depleted Cu^2+^ concentration gradients that develop once the initial nuclei form on the Al surface.

The choice of Al as the sacrificial template is also notable in its own right: selective dissolution of Al from Cu–Al alloys is a well-established route to nanoporous, high-surface-area Cu architectures for electrochemical energy storage, underscoring that the Cu–Al reduction-potential couple exploited here is a generally useful platform for building hierarchical Cu nanostructures, not a peculiarity of this particular synthesis [21]. More broadly, galvanic replacement between a Cu template and a more noble metal salt has recently been used to build a range of bimetallic architectures—for example, nanoporous Cu/Ag triangular nanoprisms for catalytic nitrophenol reduction and Cu–Ag composite membranes for redox catalysis—illustrating that the same reduction-potential-driven mechanism responsible for the Cu NDs studied here can be extended to more complex multi-metal nanostructures when a second metal salt is introduced into the bath [22,23].

**Figure 2:**
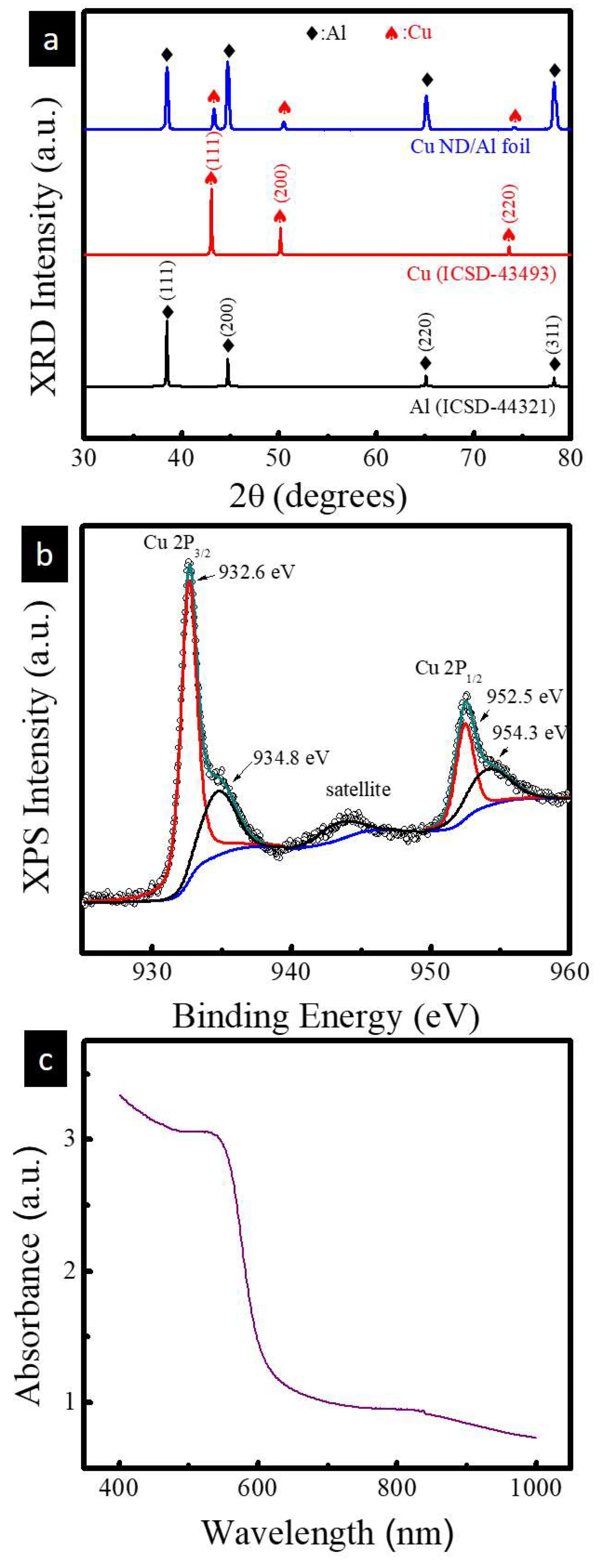
(**a**) X-ray diffraction pattern, (**b**) Cu 2p XPS spectrum, and (**c**) UV–vis absorption spectrum of the Cu NDs/Al foil sample.

The crystal structure of the Cu NDs/Al foil sample was examined by XRD (Figure 2a), with reference ICSD patterns for Cu and Al included for comparison. All diffraction peaks index to the (111), (200), and (220) reflections of face-centered-cubic Cu and Al (space group Fm-3m, ICSD 43493); no additional peaks attributable to copper or aluminum oxides were resolved, indicating that oxide formation was largely avoided during synthesis. XPS confirms this assignment: the Cu 2p spectrum (Figure 2b) shows peaks at 932.6 and 952.5 eV corresponding to the Cu 2p_3/2_ and 2p_1/2_ core levels of metallic Cu(0), with only a minor contribution from surface CuO arising from mild atmospheric oxidation. These XRD and XPS results together confirm that the Cu NDs are predominantly metallic, which is important because surface oxidation is known to damp the free-electron plasma responsible for LSPR and would be expected to weaken both the SERS and PEC responses discussed below. The UV–vis absorption spectrum (Figure 2c) shows an SPR band centered near 570 nm. This value is essentially identical to the SPR position reported for colloidal Cu nanoparticles synthesized by wet-chemical reduction, for which the same absorption feature near 570 nm has been assigned specifically to the LSPR of metallic Cu(0) rather than to any Cu oxide phase [4], reinforcing the XRD/XPS conclusion that the as-grown Cu NDs/Al foil sample is predominantly metallic. The visible-range position and considerable width of this absorption band reflect the hierarchical, multi-scale morphology of the Cu particles: a distribution of branch diameters and junction geometries supports a broad envelope of local plasmon resonances rather than the single sharp peak expected of a monodisperse nanoparticle ensemble, and it is this broad, structurally tunable absorption that is subsequently exploited for both SERS and PEC sensing.

### 3.2. SERS Performance

**Figure 3:**
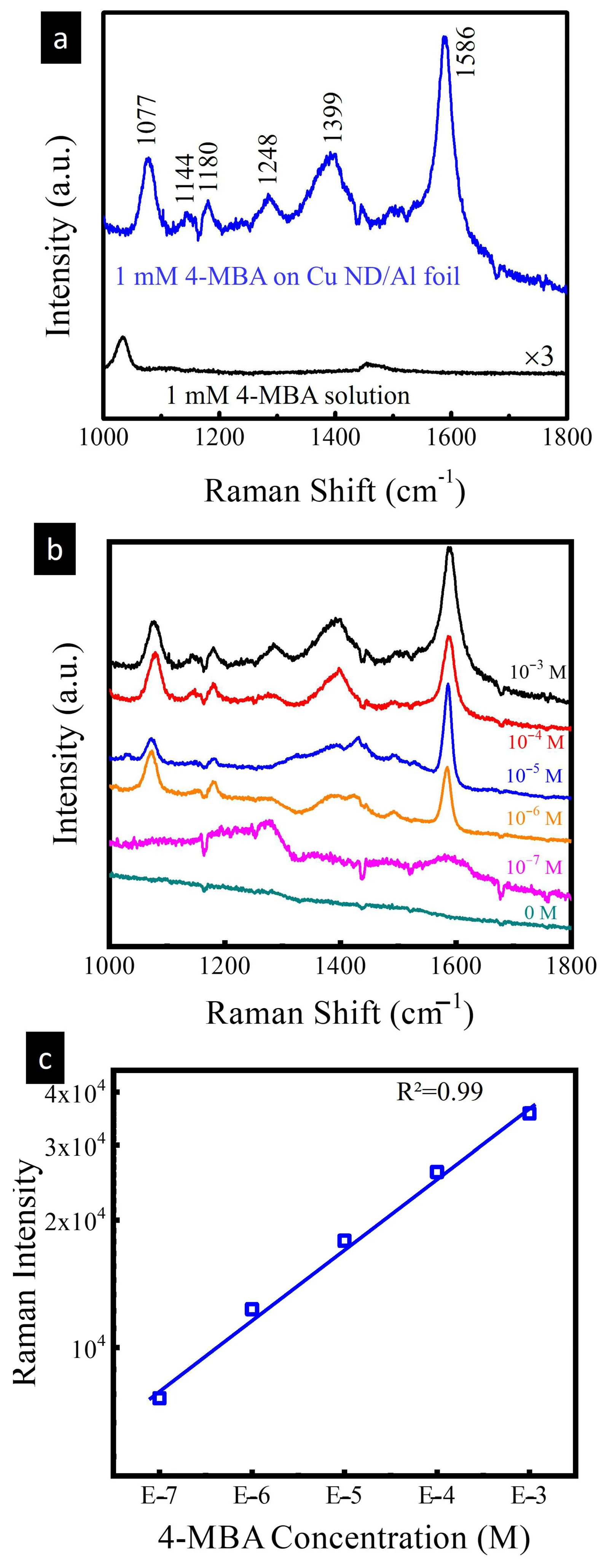
(**a**) Raman spectra of 4-MBA in solution and adsorbed on Cu NDs/Al foil. (**b**) Raman spectra of 4-MBA at varying concentration adsorbed on Cu NDs/Al foil. (**c**) Linear relationship between the logarithmic intensity at 1589 cm^−1^ and 4-MBA concentration.

Figure 3a compares the Raman spectrum of 1 mM 4-MBA in solution with that of 4-MBA adsorbed on the Cu NDs/Al foil substrate. Characteristic 4-MBA SERS bands are observed at 1080, 1143, 1181, 1282, 1402, and 1588 cm^−1^: the 1080 cm^−1^ band is assigned to the ν_8a_(a_1_) aromatic ring mode coupled with C–S stretching, and the 1588 cm^−1^ band to the ν_12_(a_1_) ring-breathing mode; the weaker 1403 cm^−1^ band corresponds to ν(COO^−^) stretching—a mode whose intensity is known to be sensitive to the protonation state of the carboxylic acid group and has been used elsewhere to probe local pH and pKa at plasmonic metal–electrolyte interfaces [24]—and the 1142 cm^−1^ (ν_15_, b_2_) and 1182 cm^−1^ (ν_9_, a_1_) bands to C–H deformation modes. These assignments confirm that the 3D Cu NDs/Al foil substrate provides substantial Raman enhancement, consistent with the electromagnetic (Cu-to-molecule) mechanism established for 4-MBA and other aromatic thiols adsorbed on Cu-based nanomaterials [4]. The magnitude of this enhancement is closely tied to substrate morphology: fern-like, multiply-branched metal architectures grown directly on a conductive metal support—whether by electroless deposition of Ag nanodendrites on Cu, templated electrodeposition of three-dimensional dendritic Ag nanostructures, or, as here, galvanic-replacement growth of Cu NDs on Al—have consistently been shown to concentrate electromagnetic hot spots at branch tips, side-branch junctions, and inter-dendrite gaps far more effectively than smooth films or isolated nanoparticles, and this hot-spot density is the key structural feature that pushes SERS detection limits down toward the trace and ultra-trace regime for both organic dyes and biomolecular probes [6,7]. For instance, electroless deposition of silver nanodendrites onto copper plates has been reported to detect the dye Rhodamine 6G down to 10^−11^ M in water, while templated three-dimensional dendritic Ag nanostructures with additional branch-junction hot spots have amplified the Raman signal of lactic acid by roughly five orders of magnitude relative to a non-SERS-active surface [6,7]. The performance of the present Cu NDs/Al foil substrate, which resolves 4-MBA down to 10^−7^ M with an estimated approximate enhancement factor of ≈10^6^, is therefore consistent in order of magnitude with these dendritic Ag/Cu benchmarks, while relying entirely on an earth-abundant, non-noble metal and a single, additive-free growth step.

To determine the detection limit, samples were immersed in 4-MBA solutions ranging from 0.1 to 10^−7^ M (Figure 3b); the SERS signal decreased monotonically with decreasing concentration, and clear 4-MBA signatures remained detectable down to 10^−7^ M. To further verify the reliability of the SERS response at the lowest concentration, additional measurements were performed at different positions on the Cu NDs/Al foil substrate using a 4-MBA concentration of 10^−7^ M. The characteristic Raman bands of 4-MBA were reproducibly detected at all examined positions, confirming that the observed signals were not limited to a specific measurement location. Accordingly, 10^−7^ M is established as the experimentally verified lowest detectable concentration under the measurement conditions employed. This detection limit compares favorably with those reported for other low-cost, non-noble or dendritic SERS platforms operating on comparable probe molecules, and is consistent with the general observation that hierarchical branching—rather than the specific identity of the plasmonic metal—is the dominant factor controlling ultimate sensitivity in this class of substrate [6,7]. A linear relationship (Figure 3c) was obtained between the logarithm of the 4-MBA concentration and the intensity of the 1588 cm^−1^ fingerprint peak:

log I = 49,898 + 6164.9 log C(1)

where I is the SERS peak intensity and C is the 4-MBA concentration. Using 4-MBA as the probe molecule, the approximate enhancement factor (EF) of the Cu NDs/Al foil substrate was estimated from the standard SERS enhancement-factor formalism used for Cu-based substrates [4]:

EF = (I_SERS_/I_bulk_)(N_bulk_/N_ads_) (2)

where I_SERS_ and I_bulk_ are the Raman intensities at 1588 cm^−1^ for 4-MBA adsorbed on the Cu NDs and for 4-MBA in solution, respectively, and N_ads_ and N_bulk_ are the corresponding numbers of probed molecules on the substrate and in the bulk solution within the laser spot. N_bulk_ and N_ads_ were estimated from the laser spot radius (≈5 μm) and penetration depth (≈15 μm), giving N_bulk_/N_ads_ ≈ 10^4^. The corresponding intensity ratio I_SERS_/I_bulk_ was ≈10^2^, yielding an EF of ≈10^6^. This approximate enhancement factor confirms that the 3D Cu NDs/Al foil substrate is an effective platform for trace-level SERS detection. It is worth noting that this bulk-averaged EF definition, while standard practice and adopted here for comparability with the earlier Cu-nanostructure literature, is known to have limitations when applied to colloidal or highly heterogeneous plasmonic aggregates; more refined figures of merit that explicitly account for the active analyte concentration contributing to the SERS signal have been proposed to address these limitations, and could be applied to the present Cu NDs/Al foil system in future work for a more rigorous comparison across substrate types [25]. To further assess the reliability of the SERS measurements, the Raman spectra were re-measured, and the characteristic Raman bands of 4-MBA were consistently reproduced. These results support the reproducibility of the SERS response obtained using the Cu NDs/Al foil substrate. Nevertheless, localized heating may occur under focused laser irradiation, potentially affecting the molecular structure or adsorption state of 4-MBA. Therefore, laser-induced molecular changes cannot be completely excluded under the present measurement conditions (5 mW laser power and 30 s accumulation time). Further systematic studies involving different laser powers and exposure times are required to clarify the possible contribution of such effects.

### 3.3. LSPR-Assisted Photoelectrochemical Detection of H_2_O_2_

**Figure 4:**
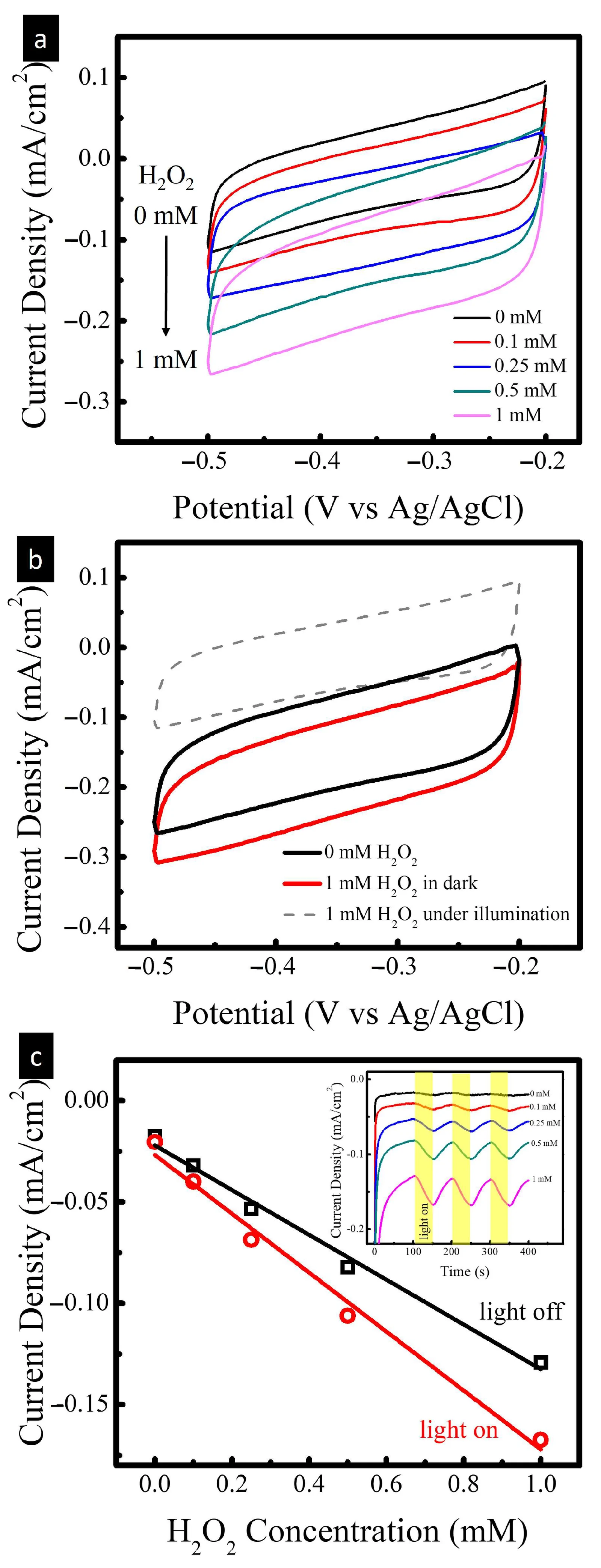
CV curves of the Cu NDs/Al foil electrode in PBS with and without H_2_O_2_ (**a**) in the dark and (**b**) under illumination. (**c**) Calibration curves of the H_2_O_2_ sensor with (red) and without (black) LSPR excitation. Inset: I–t curves for H_2_O_2_ reduction at varying H_2_O_2_ concentrations under periodic on/off illumination.

The electrocatalytic behavior of the Cu NDs/Al foil electrode toward H_2_O_2_ was investigated by cyclic voltammetry (CV) over the potential window −0.5 to −0.2 V at 50 mV s^−1^. Figure 4a shows CV curves in PBS with and without H_2_O_2_ (0.1–1 mM). No redox signal was observed in PBS alone, aside from a small double-layer charging current; upon addition of H_2_O_2_, the cathodic current increased markedly, reflecting the electrocatalytic reduction of H_2_O_2_ at the Cu surface. The enzyme-free reduction mechanism on the Cu cathode is proposed to proceed as follows:

H_2_O_2_ + e^−^ → OH_ads_ + OH^−^ (3)

OH_ads_ + e^−^ → OH^−^ (4)

As the H_2_O_2_ concentration was raised to 1 mM, the cathodic current density on the Cu NDs/Al foil electrode increased accordingly. Under simulated solar illumination, this cathodic current was further enhanced relative to the dark condition (Figure 4b), consistent with the injection of LSPR-generated hot electrons from the Cu NDs into the H_2_O_2_ reduction pathway. This effect is also evident in the steady-state photocurrent recorded at a fixed potential of −0.4 V (Figure 4c): the cathodic current rises sharply when the LSPR excitation is switched on and decays immediately when the light is switched off. Calibration curves derived from these measurements (Figure 4c) show a linear response up to 1 mM H_2_O_2_, with a detection sensitivity of 110.6 μA cm^−2^ mM^−1^ in the dark, increasing to 145.3 μA cm^−2^ mM^−1^ under LSPR excitation—an enhancement of approximately 31%. These results demonstrate that LSPR excitation of low-cost Cu nanostructures can meaningfully improve the sensitivity of enzyme-free electrochemical H_2_O_2_ sensing, placing the Cu NDs/Al foil electrode within the broader family of plasmon-enhanced H_2_O_2_ sensors recently reported using noble-metal nanostructures. For comparison, Pd-tipped Au nanobipyramids generate high-energy electron–hole pairs under LSPR excitation that substantially enhance the electrochemical reduction of H_2_O_2_, achieving high sensitivity and a low detection limit suitable for monitoring extracellular H_2_O_2_ from live cells [11], while size-tunable triangular Ag nanoplates deposited on screen-printed carbon electrodes show a H_2_O_2_ reduction sensitivity that scales with nanoplate aspect ratio and SPR peak position, directly linking plasmonic tunability to sensing performance [12]. More recently, a core–shell Au nanobipyramid–MoS_2_ hybrid electrode exploiting plasmon–semiconductor coupling achieved an order-of-magnitude photocurrent enhancement for nonenzymatic H_2_O_2_ and glucose sensing under resonant near-infrared excitation [13]. The present Cu NDs/Al foil system achieves a qualitatively similar LSPR-driven enhancement using an earth-abundant, non-noble metal and a single-step synthesis, underscoring that the underlying hot-carrier injection mechanism is not restricted to gold- or silver-based nanostructures. The present result is also consistent with the broader, non-plasmonic literature on Cu-based enzyme-free H_2_O_2_ sensors: nanostructured CuO petals, CuO/polymer nanocomposites, and Ag-Cu nanoalloys have all been reported as effective non-enzymatic H_2_O_2_ electrocatalysts in the dark, with sensitivities in the same general range as the dark-state value measured here, while Ag-Pt core–shell nanowires illustrate a complementary strategy of stabilizing a plasmonic metal against oxidation to preserve H_2_O_2_-sensing performance over time [26,27,28,29]. The minor surface CuO detected by XPS is not expected to dominate the sensing response. It may provide auxiliary catalytic sites and contribute to the dark catalytic current; however, the LSPR absorption and the photocurrent action spectrum both correlate with metallic Cu(0), indicating that the plasmon-enhanced PEC response originates mainly from the Cu NDs. The oxide contribution is therefore considered secondary and does not alter the main conclusion. Mechanistically, the enhancement observed here is consistent with studies that have specifically examined hot-electron injection from Cu nanostructures rather than from Au or Ag: Cu nanoparticles anchored on semiconductor supports have been shown to inject near-infrared-generated hot electrons across a Cu–support interface to drive photocatalytic reduction reactions, and morphology-dependent studies of plasmonic hot-electron generation and injection have shown that nanostructure geometry—branching, tip curvature, and junction density in particular—can be tuned to improve carrier extraction efficiency, a consideration directly relevant to the dendritic morphology of the Cu NDs studied here [30,31]. Although the limit of detection was not specifically optimized in this work, the sensitivity of the Cu NDs/Al foil electrode under LSPR excitation (145.3 μA mM^−1^ cm^−2^) compares favorably with previously reported non-noble Cu-based H_2_O_2_ sensors (Table 1) [32,33,34,35,36,37]. This demonstrates that the LSPR-generated hot-carrier injection effectively boosts the electrocatalytic reduction of H_2_O_2_, making this low-cost, earth-abundant Cu nanodendrite platform a promising candidate for sensitive PEC sensing.

**Figure 5:**
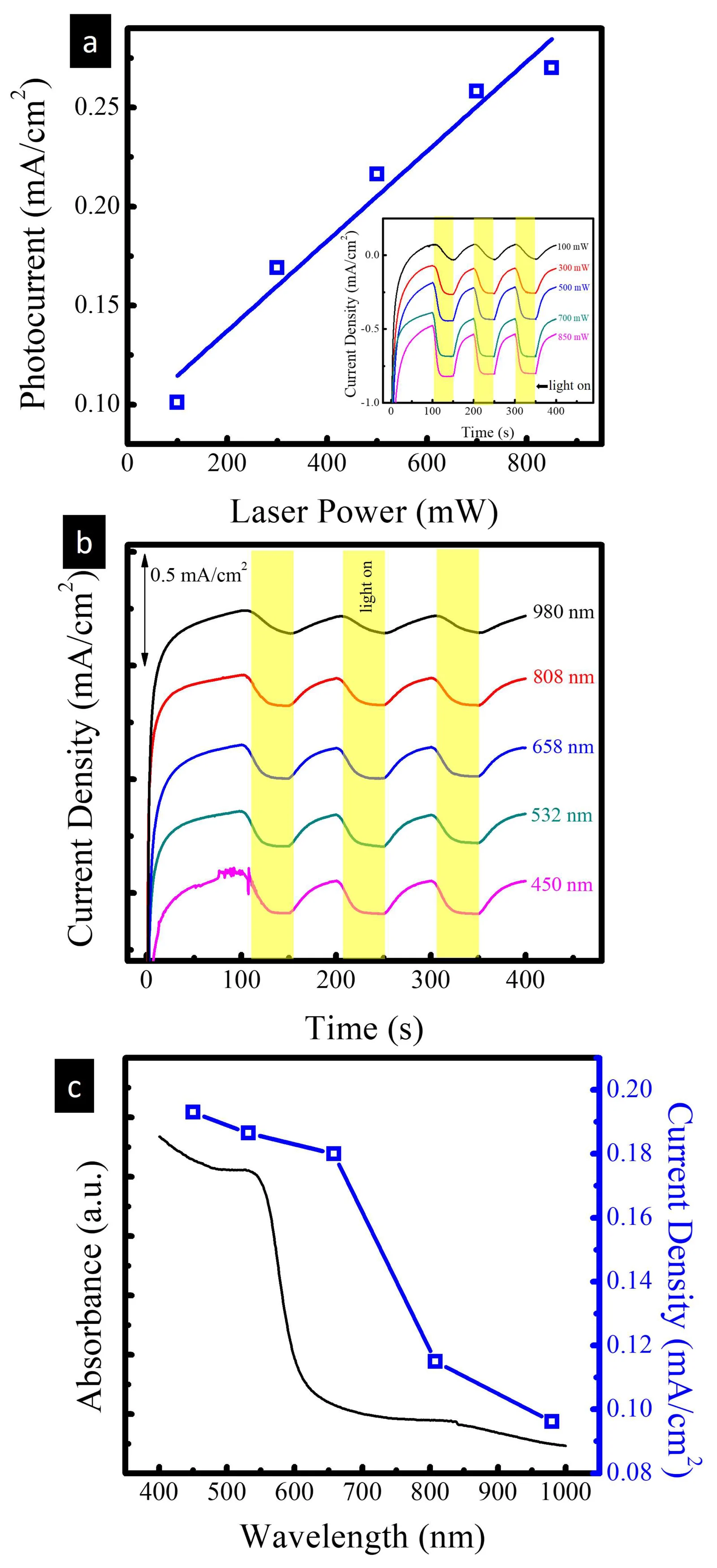
(**a**) Peak current at −0.4 V for electrocatalytic reduction of 1 mM H_2_O_2_ as a function of 450 nm illumination intensity. (**b**) I–t curves at five different excitation wavelengths. (**c**) Wavelength-dependent peak current for H_2_O_2_ reduction (blue) and LSPR absorption spectrum (black) of the Cu NDs.

To further probe the LSPR contribution, the dependence of the photocurrent on illumination intensity and wavelength was examined. Figure 5a shows that the LSPR-enhanced H_2_O_2_ reduction current at −0.4 V increases linearly with the intensity of 450 nm illumination, mirroring the intensity dependence previously reported for a plasmonic Ag nanowire/carbon cloth electrode under simulated solar light. This linear dependence rules out a dominant contribution from photothermal heating and instead points to hot-carrier generation associated with the photoexcitation of the Cu NDs. This interpretation is consistent with the current understanding of plasmonic hot-electron dynamics, in which absorbed photon energy is first converted into a non-equilibrium distribution of energetic carriers well above (or below) the Fermi level before these carriers thermalize with the lattice; because hot-carrier lifetimes are typically limited to femto- to picosecond timescales, only carriers generated close to the metal surface, and with sufficient excess energy to overcome the relevant reaction barrier, can be extracted before relaxing to heat [8,9,10]. A linear dependence of photocurrent on illumination intensity is a signature commonly used to distinguish this direct hot-carrier injection pathway from photothermally driven catalysis, for which the current would instead scale with the (generally sublinear) temperature rise of the electrode. Wavelength-dependent measurements of the steady-state H_2_O_2_ reduction current at −0.4 V (Figure 5b) reveal a peak response that is red-shifted relative to the LSPR absorption band of the Cu NDs (Figure 5c), which can be attributed to changes in the local refractive index around the Cu NDs associated with surface-adsorbate complex formation. Aside from this shift, the wavelength dependence of the photocurrent closely tracks the LSPR absorption profile, confirming that LSPR excitation—rather than direct band-gap or thermal effects—is the primary driver of the enhanced cathodic current for H_2_O_2_ reduction on Cu NDs. This wavelength-tracking behavior parallels observations for other plasmon-enhanced PEC sensing platforms, where the photoresponse of the device follows the LSPR absorption profile of the active nanostructure rather than that of any accompanying semiconductor or support material, reinforcing the assignment of LSPR excitation as the dominant enhancement pathway [9]. The enhanced photocurrent under illumination is strongly associated with the LSPR response of the Cu NDs. The photocurrent increases linearly with 450 nm illumination intensity, while the wavelength-dependent action spectrum closely follows the Cu-ND LSPR absorption feature. In addition, the measured surface temperature increase under illumination is only 0.2 °C. These observations collectively support an LSPR-mediated hot-carrier contribution as the dominant origin of the enhanced H_2_O_2_ reduction response under the present conditions. Nevertheless, a minor contribution from localized photothermal effects cannot be completely excluded. Beyond electrochemical sensing, plasmon-enhanced electrochemical imaging approaches—for example, Prussian blue nanofilms sensitized for plasmonic electrochemical microscopy—have recently been used to visualize the spatially localized delivery of H_2_O_2_ at a single-electrode level [14], suggesting that the present Cu NDs/Al foil platform could, in principle, be extended from bulk amperometric sensing toward spatially resolved or imaging-based H_2_O_2_ detection in future work. Finally, the general strategy of growing dendritic, high-surface-area Cu-based bimetallic nanostructures by facile electrodeposition or galvanic routes continues to expand into adjacent electrocatalytic applications, such as three-dimensional dendritic Cu–Sn alloys recently reported for the electrocatalytic reduction of nitrate to ammonia, suggesting that the synthetic approach demonstrated here could be adapted to other dendritic Cu-based bimetallic systems for electrocatalysis beyond sensing [32]. In this study, a “synthesize-and-measure-immediately” protocol was adopted to minimize the time between Cu-ND synthesis and sensing measurements. After galvanic replacement, the electrodes were quickly rinsed and immediately transferred either to a 4-MBA solution for SERS measurements or to PBS for electrochemical/PEC measurements. During these procedures, direct exposure of the Cu-ND surface to air was minimized. The adsorbed 4-MBA layer and the electrolyte environment are expected to retard surface oxidation and reduce potential oxidation-related interference during measurement. In addition, independently prepared Cu-ND/Al foil electrodes exhibited reproducible SERS intensities at 1588 cm^−1^ and PEC sensitivities toward H_2_O_2_. These results support the functional reproducibility of the electrodes under the immediate-use protocol. Nevertheless, quantitative analysis of the Cu surface oxidation states after measurement and long-term aging studies were not performed; therefore, changes in surface chemical states and long-term storage stability cannot be conclusively evaluated.

## 4. Conclusions

Hierarchical Cu NDs were grown on Al foil in a single step via galvanic replacement and evaluated as a dual-function plasmonic sensing platform. SEM, XRD, and XPS confirmed a fern-leaf-like, predominantly metallic Cu morphology. As a SERS substrate, the Cu NDs/Al foil sample achieved ultrasensitive detection of 4-MBA down to 10^−7^ M with an approximate enhancement factor of ~10^6^. As an enzyme-free PEC H_2_O_2_ sensor, the same electrode showed a linear response to H_2_O_2_ concentration with good reproducibility, and its sensitivity increased by approximately 31% under simulated solar illumination relative to the dark. Intensity- and wavelength-dependent measurements confirm that this enhancement originates from LSPR-generated hot carriers rather than photothermal effects. Together, these results establish low-cost, earth-abundant Cu nanodendrites as an effective platform for combining optical and electrochemical (bio)sensing, and point toward broader opportunities in plasmon-enhanced electrochemical device design.

## Figures and Tables

**Table 1:** Comparison with literature regarding the performance of H_2_O_2_ sensing.

Sensor Type	Detection Limit (μM)	Linear Range (mM)	Sensitivity (μA mM^–1^ cm^–2^)	Ref.
Cu_2_O/GN	20.8	0.3–7.8		[33]
ZnO_3_–CuO_7_/CPE	2.4	0.003–0.53	1.11	[34]
CuO–SiNWs/GCE	1.6	0.01–13.18	22.27	[35]
CuO nanoflowers	5	0.05–4.600	116.1	[36]
CuCo_2_O_4_	3.0	0.01–8.90	94.1	[37]
Cu ND (light)	none	0.1–1	145.3	This work

## Data Availability

The data are contained within the article.
